# Comprehensive Insight into Gibberellin- and Jasmonate-Mediated Stamen Development

**DOI:** 10.3390/genes10100811

**Published:** 2019-10-15

**Authors:** Katarzyna Marciniak, Krzysztof Przedniczek

**Affiliations:** Chair of Plant Physiology and Biotechnology, Institute of Biology, Faculty of Biological and Veterinary Sciences, Nicolaus Copernicus University, Lwowska 1 St, 87-100 Toruń, Poland; krzysztofprzedniczek@gmail.com

**Keywords:** gibberellins, jasmonates, stamen development, phytohormone interactions

## Abstract

In flowering plants, proper development of male generative organs is required for successful sexual reproduction. Stamen primordia arise in the third whorl of floral organs and subsequently differentiate into filaments and anthers. The early phase of stamen development, in which meiosis occurs, is followed by a late developmental phase, which consists of filament elongation coordinated with pollen maturation, anther dehiscence and finally viable pollen grain release. Stamen development and function are modulated by phytohormones, with a key role of gibberellins (GAs) and jasmonates (JAs). Long-term, extensive investigations, mainly involving GA/JA-deficient and GA/JA-response mutants, have led to a better understanding of the hormone-dependent molecular mechanisms of stamen development. In several species, the principal functions of GAs are to stimulate filament elongation through increased cell elongation and to promote anther locule opening. In the GA-dependent regulation of early stamen development, both the tapetum and developing pollen were identified as major targets. JAs mainly control the late stages of stamen development, such as filament elongation, viable pollen formation and anther dehiscence. A hierarchical relationship between GAs and JAs was recognized mainly in the control of late stamen development. By repressing DELLA proteins, GAs modulate the transcriptional activity of JA biosynthesis genes to promote JA production. A high level of JAs induces a complex of transcription factors crucial for normal stamen development.

## 1. Introduction

**Figure 1:**
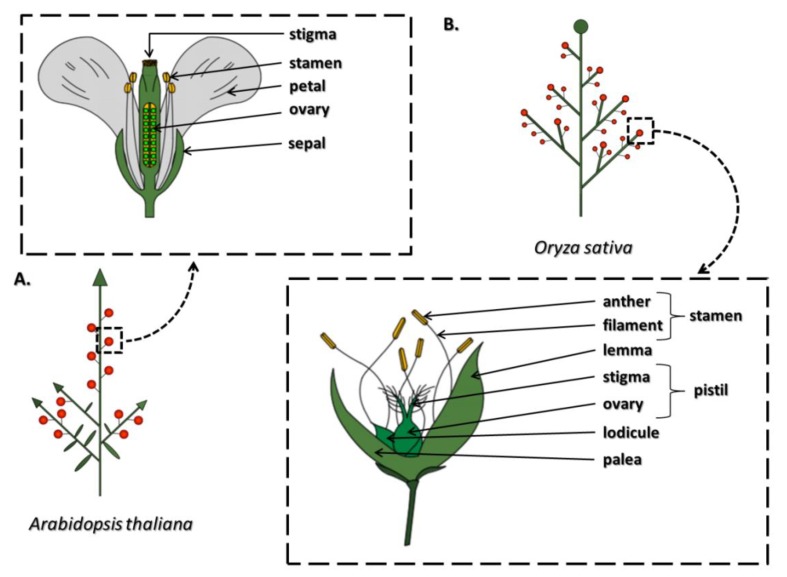
The inflorescence architecture and flower structure in the model dicotyledonous species *Arabidopsis thaliana* (**A**) and in the model monocotyledonous species *Oryza sativa* (rice) (**B**) According to [1,3,4,5].

The proper formation, development, and functioning of generative organs in plants are crucial for maintaining the continuity of a species. A flower is a contracted stem with limited growth whose particular elements have undergone modifications to optimize the requirements of the reproduction process (Figure 1). The basic flower construction scheme assumes the existence of four concentric whorls generated by different types of organs. In the model dicotyledonous species *Arabidopsis thaliana*, the flower consists of four sepals, four petals, six stamens, and one pistil (Figure 1A), whereas in the model monocotyledonous species *Oryza sativa* (rice), each floret typically consists of a central pistil, six stamens, and two lodicules (petals) subtended by the palea (an inner bract) and the lemma (the outer bract) (Figure 1B) [1]. However, divergence from these patterns occurs, for example, in the identity of particular elements, the order of whorl occurrence, changes in the number of whorls and their component elements, and the formation of completely new structures [2].

As a result of research conducted in the 1990s, mainly on *A. thaliana* and *Antirrhinum majus*, a model illustrating the genetic regulation of the formation of specific structures within the flower was generated. The model assumes the dependence of the identity of successive constitutive flower elements on the interaction of three classes of homeotic genes (class A, B, C), i.e., genes whose expression in a specific time and place regulates the activity of other genes in a cascade manner, resulting in a phenotype characteristic of a given whorl [6]. Further research into the ABC model has contributed to broadening of the model with further gene classes, i.e., D and E [2]. This extension made it possible to specify the mechanisms of interaction of gene activity products belonging to the ABCDE model. The floral quartet model (FQM) specifies that the identity of various floral organs is determined during development by tetrameric, MIKC-type MADS-domain (MCM1, AGAMOUS, DEFICIENS, and SRF, serum response factor) protein complexes [7]. These quartets are considered to function as transcription factors by binding to the CC(A/T)(6)GG (CArG-box) promoter sequences of their target genes. An increase or decrease in the gene expression level controls the proper development of individual floral organs [8].

In addition to well-known genetic control mechanisms, a significant role in the regulation of flower morphogenesis is played by phytohormones, i.e., gibberellins (GAs), jasmonates (JAs), auxins, brassinosteroids (BRs), and cytokinins (CKs), whose presence and appropriate balance are indispensable for the proper development of these structures [9]. The regulation of the size of the floral meristem involves CKs, GAs, and auxins. Auxins also play a leading role in organ initiation and organogenesis. The development of individual floral organs is controlled by these various hormones, e.g., auxins play crucial roles in gynoecium development, and together with GAs and JAs, in petal development. However, stamen development requires the presence of almost all phytohormones [10]. Additionally, the determination of floral sex in monoecious and dioecious plants is controlled by the auxin-GA balance, which was discovered nearly half a century ago. In many plants, the use of exogenous auxins favors the generation of a pistil, while the stamens will be defective. On the other hand, GAs contribute to stamen development [11]. Furthermore, Pharis et al. [12] showed that in forming flowers, the GA effect on sex differentiation depends on its type. Treatment of six-year-old *Pseudotsuga menziesii* seedlings with a GA_4/7_ + GA_9_ mixture increases the production of female flowers, while for the formation of male flowers, GA_4/7_ was found to be the most effective [12,13].

The results of physiological studies conducted over the years have shown pleiotropic effects of plant hormones, which led to the conviction among many plant physiologists that they are non-specific. However, significant progress in scientific research resulting mainly from the development of modern techniques of molecular biology, genetic engineering or multi-omics approaches made it possible to precisely understand the metabolic pathways as well as the perception and signal transduction pathways of plant hormones [14,15]. Then, it became clear that the action of phytohormones is specific because each of them has a distinct and definite receptor, and the initiated signaling pathway leads to the activation or repression of a characteristic set of genes and proteins, including those related to the metabolism and signaling pathways of other phytohormones. This situation is indicative of numerous interactions between plant hormones, whose varying concentrations and activities at specific times and places determine the induction of a particular physiological process [14].

The main purpose of this article is to comprehensively present the current state of knowledge about the structure of stamens and the molecular mechanisms of their development and functioning modulated by GAs and JAs. The interactions of these phytohormones are also described in detail. Due to the multitude of identified mutants, their phenotypes and functions in anther/pollen and filament development are described in separate sections, first addressing the biosynthesis and then the signaling pathways of each phytohormone. Many years of research have proven that the participation of GAs and JAs is essential during both the early and late phases of stamen development. It is assumed that during the early phase, primordia appear, all types of tissues are formed and microsporogenesis occurs. In turn, during the late phase, pollen grains mature, and anther tissue degenerates, resulting in anther dehiscence and, finally, pollen grain release. During this stage, stamen filaments elongate rapidly [16]. The high similarity between the different stages of stamen development in various plants, including rice and *A. thaliana*, as well as the high homology between the transcription factors involved in this process suggest the existence of a highly conserved program of the development for male sex organs in angiosperms [3]. Deficiency of GAs or JAs, caused by biosynthesis gene mutation, the results in the accumulation of signaling pathway repressors, which is one of the causes of phenotypic stamen abnormalities and often leads to male sterility. This affects the course of the pollination and fertilization processes and, consequently, the plant’s reproductive success [9].

## 2. Structure, Function and Development of Stamens

**Figure 2:**
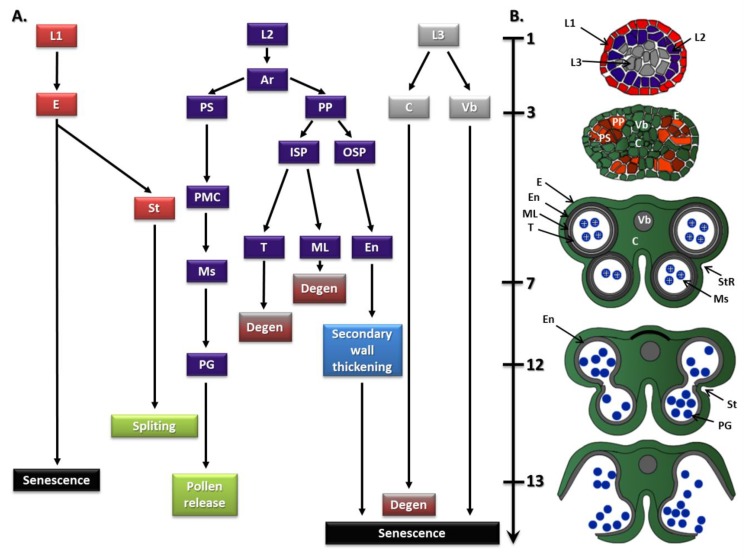
The cell lineages model for the origin of the cell layers in the anther (**A**) and anther structure at different stages of development (**B**) in *A. thaliana*. Initially, divisions in the L1, L2, and L3 layers of the floral meristem lead to the formation of the stamen primordia. Divisions in the L1 layer form the epidermis (E), stomium region (StR), and stomium (St), while L3 cells divide to form connective (C) and vascular bundle (Vb). Periclinal divisions of the L2 cells result in the formation of archesporial cells (Ar). Next, Ar divide to form the primary (1°) sporogenous layer (PS) and the primary (1°) parietal layer (PP). Then, the PS layer undergoes divisions to form pollen mother cells (PMC), microspores (Ms) and, finally, mature pollen grains (PG). The PP layer goes through a further division to form two secondary (2°) parietal layers, the inner secondary parietal layer (ISP) and the outer secondary parietal layer (OSP). The OSP divides again and differentiates to form the endothecium (En), whereas the ISP divides and develops to form the tapetum (T) and middle layer (ML) [17,18,19,20].

Anthers and filaments, as morphologically different components of the stamen, have a highly specialized structure due to their function. The anther has a bilateral structure with four locules where pollen matures (Figure 2). The tissues adjacent to each locule are the tapetum, middle layer, endothecium, and epidermis. All of these tissues participate in the protection, maturation, and release of pollen grains. A vascular bundle surrounded by connective tissue is found in the middle of the anther. The filament has a concentric structure with a centrally located vascular bundle. The main roles of this part of the stamen include not only the transport of water and nutrients but also the provision of mechanical support for the anthers [16,17,18]. 

Stamen formation occurs in the third whorl as a result of BCE class gene activity (ABCDE model). According to the FQM, a complex of SEPALLATA (SEP; the class E protein), APETALA3 (AP3; the class B protein), PISTILLATA (PI; the class B protein), and AGAMOUS (AG; the class C protein) determines stamen identity [8]. The structure of the stamens has been thoroughly described in *A. thaliana*, where 15 phases have been distinguished (Figure 2).

Starting from floral stage 5 [21,22], which is the first stage of anther development [19], the division activity of the L1, L2, and L3 layers of the meristem leads to the formation of the stamen primordia (Figure 2A). L1 cells divide and differentiate into epidermis; L3 cells give rise to vascular and connective tissues; and L2 cells give rise to archesporial cells (anther stage 2; floral stages 5–6). Periclinal divisions of archesporial cells take place in the four corners of the anther, leading to the formation of distinct primary (1°) sporogenous and primary (1°) parietal cell lineages. Next, the 1° sporogenous cells undergo a number of divisions to form pollen mother cells, whereas the 1° parietal cells go through a further division to form two secondary (2°) parietal layers, the inner secondary (2°) parietal layer and the outer secondary (2°) parietal layer. The outer secondary parietal layer then divides again and differentiates to form the endothecium, whereas the inner secondary parietal layer divides and develops to form the tapetum and middle cell layer (anther stage 4; floral stage 8). The final structure results in the gametophytes being surrounded by a series of cell layers with the following order: tapetum, middle layer, endothecium, and epidermis. In the sixth stage (floral stage 9), meiosis begins, the middle layer degenerates, the tapetum vacuolates, and the whole anther expands. After meiosis, the tetrads of haploid microspores are generated (anther stage 7; floral stage 9), and at this time, the early stage of stamen development is complete [3,17,18,19,20,23].

**Figure 3:**
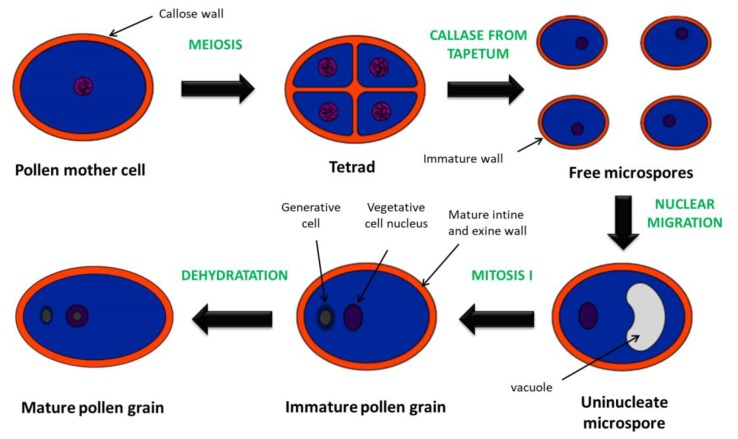
Main events during microsporogenesis of most angiosperms. Pollen mother cells undergo meiosis to form a tetrad. Individual microspores are released by the action of the callase from tapetum. Then, uninucleate microspores undergo mitosis I to form pollen with a larger vegetative cell and a smaller generative cell [17,24].

At the beginning of late stamen development, the callose wall surrounding the tetrads degenerates resulting in the release of individual microspores (anther stage 8; floral stage 10) (Figure 3). At the next stage, the microspores form exine walls and are vacuolated (anther stage 9; floral stage 10). The tapetum degenerates, followed by the first pollen mitotic division (anther stage 10–11; floral stage 11–12). The second mitotic division ends the process of pollen maturation, forming tricellular pollen grains (anther/floral stage 12) [17,19]. 

Expansion of the endothecium, which is part of the extensive dehiscence program, takes place around stage 11 of anther development (floral stage 11–12). Both the endothecium and connective tissues deposit ligno-cellulosic fibrous bands during the secondary thickening process. Then, the septum degenerates, generating bilocular anthers at anther/floral stage 12. The loosening of stomium cells facilitates the breakdown of these highly specialized epidermal cells, leading to anther opening and pollen release (anther stage 13; floral stage 13–14). The stamen filament grows rapidly during dehiscence due to cell elongation but not an increase in the number of cells. At anther stage 13 (floral stage 14), further extension of filaments occurs which allows the stigma to be avoided. Finally, anther senescence occurs (anther stage 14; floral stage 15–16), and the anther then falls off the plant (anther stage 15; floral stage 17) along with the rest of the flower [16,19].

The above description shows that much information about anther and pollen development in the dicotyledonous species *A. thaliana* is now available. Nevertheless, significant progress has also been made in the elucidation of anther/pollen development in monocotyledonous plants, including rice [3,25]. Pollen formation with the development of a secretory tapetum in rice is very similar to that observed in *A. thaliana* [26,27]. A number of male sterile mutants have been recognized during rice pollen development, revealing a high degree of conservation in the early regulatory network of pollen formation [3]. To deepen knowledge about anther/pollen development pathways in *A. thaliana*, rice and also anther crop species we recommended the references [3,23,25].

In conclusion, stamen development in most angiosperms involves a complex, coordinated, and synchronized interaction between sporophytic and gametophytic tissues. This leads to the development of functional pollen, its release, and consequently, pollination, fertilization, and seed set. The application of different information (mainly about male sterile mutants) regarding anther development to other species, particularly cereals, will provide opportunities to control fertility in economically important crops (wheat, barley) in which the reproduction process is currently less well understood [23].

## 3. Gibberellin-Mediated Stamen Development and Functioning

Gibberellins are phytohormones that are mainly studied regarding their participation in the process of flowering induction in plants with various photoperiodic sensitivities [13,28,29,30]. Although this research has been carried out in a limited number of species, it seems that GAs are not a universal flowering stimulus, and it is assumed that they act in a species-specific manner. In biennial and long-day plants (LDPs), GAs usually promote the transition from vegetative to generative development. In *A. thaliana*, which is a classic example of an LDP, GAs have a minor influence on flowering time under inductive, long-day (LD) conditions, while in the absence of the appropriate photoperiod during short-day (SD) conditions, GAs are obligatory [30]. On the other hand, in many short-day plants (SDPs) cultivated under inductive SD or non-inductive LD conditions, GA application delays or even inhibits flowering [13]. In *Ipomoea nil*, a model SDP, cultivated under sub-inductive conditions, GAs stimulate flower bud formation [31]. In turn, molecular and immunocytochemical analyses revealed that photoperiodic flower induction in *I. nil* is accompanied by decreasing contents of GAs [32]. Thus, the effects observed in SDPs are not unequivocal and strictly depend on the time and place of GA action. Research using labeled GA standards indicates that light is one of the factors affecting the transport of GAs from cotyledons to the epicotyl [33]. Stimulation of flowering was observed in *I. nil* seedlings without cotyledons that were treated with GAs on the apex [34]. Hence, it has been postulated that in this species the main role of GAs is to participate in the mechanism of flower evocation and morphogenesis [32,33]. It should be added that it is only in *Lolium temulentum* (LDPs) where it has been clearly observed that photoperiodic induction leads to an increase in GA levels in leaves followed by the transport of GAs to the apex as a mobile signal. In the process of flower morphogenesis, the role of GAs is more general and probably universal [30]. The GA signaling pathway is not required to differentiate individual parts of the flower, but it is the basis for the normal development of flower organs [35].

The involvement of GAs in the formation, development, and functioning of stamens is more advanced than that for the other phytohormones [1]. In *A. thaliana*, impaired male fertility resulting from abnormal stamen development occurs in mutants with even mild deficiency of GAs [36,37]. A significant or extreme reduction of GAs leads to female sterility in *A. thaliana* and *Lycopersicon esculentum* (tomato) [38,39]. Additionally, it has been confirmed that a higher concentration of GAs is required more for proper *A. thaliana* stamen development than for pistil, petal, or sepal development [38]. Both GA-deficient and GA-responsive *A. thaliana* mutants exhibit short stamens as a result of reduced cell extension within the filament, which prevents self-pollination [40]. Furthermore, appropriate GA levels are also essential for correct anther development, allowing mature and viable pollen formation, and finally dehiscence [30]. Characteristic features related to stamen development of GA-deficient and GA-responsive mutants are presented in Table 1 and Table 2, respectively.

### 3.1. GA Biosynthesis Is Crucial for Correct Stamen Development and Functioning in Various Plants

#### 3.1.1. GA Biosynthesis Pathway—General Information

**Figure 4:**
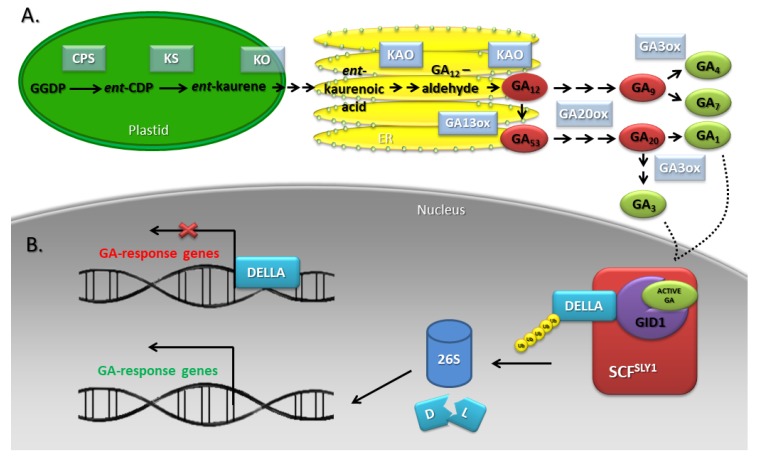
GA biosynthesis (**A**) and signaling (**B**) pathways in *A. thaliana*. GAs are synthesized in plastids, the endoplasmic reticulum (ER), and the cytosol. There are several steps in the methylerythritol phosphate (MEP) pathway: geranyl geranyl diphosphate (GGDP) is converted to *ent*-copalyl diphosphate (*ent*-CPD) by *ent*-copalyl diphosphate synthase (CPS); *ent*-CDP is converted to *ent*-kaurene by *ent*-kaurene synthase (KS); *ent*-kaurene is converted through *ent*-kaurenol, *ent*-kaurenal to *ent*-kaurenoic acid by *ent*-kaurene oxidase (KO); *ent*-kaurenoic acid is converted to GA_12_-aldehyde (through the *ent*-7a-hydroxykaurenoic acid) by *ent*-kaurene acid oxidase (KAO). GA_12_-aldehyde is converted to GA_12_ by KAO and GA_12_ to GA_53_ by gibberellin 13-oxidase (GA13ox). GA_12_ and GA_53_ are processed to the bioactive GAs by oxidations on C-20 and C-3, which is accomplished by GA 20-oxidase (GA20ox) and GA 3-oxidase (GA3ox). The binding of DELLAs by the GA-GID1 (GA INSENSITIVE DWARF1) complex increases their affinity for the SCF^SLY1^ E3 ubiquitin ligases, and polyubiquitin chain-labeled proteins are degraded by 26S proteasome. These events lead to the activation/unblocking of specific transcription factors that interact with the promoters to regulate GA-response genes. According to [9,44,45].

According to the intracellular distribution of the enzymes catalyzing subsequent reactions, three stages of the GA biosynthetic pathway have been distinguished: *ent*-kauren biosynthesis in plastids, aldehyde GA_12_ formation in the endoplasmic reticulum (ER), and synthesis of C_19_-GAs and C_20_-GAs in the cytosol (Figure 4A) [41,42]. Among the enzymes involved in the early stages of GA biosynthesis, *ent*-copalyl pyrophosphate synthase (CPS), *ent*-kaurene synthase (KS), *ent*-kaurene 19-oxidase (EKO), and *ent*-kaurenoic acid oxidase (KAO) have been distinguished. Late biosynthesis stages are catalyzed by gibberellin 20-oxidases (GA20oxs) and gibberellin 3-oxidases (GA3oxs) responsible for the synthesis of active phytohormone molecules and gibberellin 2-oxidases (GA2oxs) that inactivate GAs. Whereas early-stage enzymes occur in a single copy, late-stage enzymes are encoded by multigene families [42,43]. GA biosynthesis takes place beginning in the early stages of *A. thaliana* flower bud development. Expression of *CPS* and *GA3ox* occurs only in the receptacle and the stamen immediately after the emergence of floral organ primordia (floral stage 7). On this basis, it was concluded that these organs may be a source of GAs for other floral organs [1]. In *Petunia hybrida*, the development of the petals is dependent on the GAs originating from the stamens [30]. Furthermore, rice anthers, especially the tapetum tissue, exhibit high concentrations of GAs, whereas other floral tissues do not [1]. 

#### 3.1.2. Early Stages of GA Biosynthesis

The *A. thaliana* mutant *ga1-3*, which harbors a deletion in CPS, is male sterile. The results of anatomical analyses showed that this sterility is mainly related to inhibition of stamen filament elongation and inability to complete anthesis [40]. The shortening of the filament in *ga1-3* is caused by a reduction of only the length and not the number of cells because the mutant has a comparable content of filament epidermal cells to wild type (WT) plants. Mature mutants show an altered ratio of stamen-pistil length in the flowers, which prevents pollination and fertilization [40]. The results of investigations carried out with exogenous phytohormones revealed that the sterility of *ga1-3* can be reversed by GA application [46]. Further studies have shown that up to approximately floral stage 10, which corresponds to microsporogenesis stages 7–8 [22], all floral organs are properly initiated and develop normally in *ga1-3*. However, the following stages exhibit disorders in stamen and petal development. In this mutant, anther and pollen development is blocked after meiosis but before mitosis due to the inability to release microspores and pollen sac expansion arrest. Furthermore, the *ga1-3* tapetum remains at the vacuolated stage and degenerates together with the microspores [40]. In other plant species mutants with high GA deficiency, e.g., tomato *gib-1* (a loss-of-function mutant in copalyl diphosphate synthase, CPS) and *ga-2* (a loss-of-function mutant in kaurenoic acid oxidase, KAO), the inhibition of microsporogenesis occurs before meiosis [39,47]. Aya et al. [48] showed that *oscps1-1* (null allele of *OsCPS1*), which is one of the most severe GA-deficient mutants in rice, is characterized by abnormal enlargement of tapetal cells and collapse of microspores [48].

Another rice intermediate severity GA-deficient mutant, *reduced pollen elongation1* (*rpe1*), with defects in the male gametophyte exhibits reduced pollen germination and elongation [49]. Through the pretreatment of *rpe1* stigmas with GA_4_ before self-pollination, Chhun et al. [49] confirmed that the impaired germination and elongation of *rpe1* pollen is also caused by a deficiency of GAs and, furthermore, strictly depends on the GA concentration. Interestingly, the mutant develops typical flowers with normal pistils and stamens. It has been shown that pollen viability and the number of mature pollen grains in *rpe1* are similar to those of the WT plant. Further investigation revealed that the *rpe1* mutant phenotype is caused by a partial defect in the functioning of *OsKAO*. Subsequent expression analysis of this gene as well as *CPS1*, *KS1,* and *KO2* revealed high levels of their transcripts at the tetrad stage or later, i.e., after meiosis; however, no or low transcriptional activity of GA synthesis-related genes was observed at the premeiotic stage. Moreover, a correlation between expression patterns and the genetic frequency of transmission of mutant alleles during the process of pollen development was found [49]. This suggests that the transcriptional activity of genes around meiosis is critical in determining the transmission frequencies of mutant alleles of GA synthesis genes. 

#### 3.1.3. Late Stages of GA Biosynthesis

A study performed by Mitchum et al. [50] on the transcriptional activity of the late GA biosynthesis genes showed that the *GA3ox3* and *GA3ox4* transcripts accumulate mainly in reproductive organs [50]. Further investigations of tissue- and cell-specific expression patterns confirmed that in flowers, both genes are only expressed in anthers, but the expression of *AtGA3ox3* is much stronger than that of *AtGA3ox4*. Around the sixth stage of early anther development, the expression of these genes begins in the whole anther, although at a low level. Then, at stages 9-10, just before pollen mitosis, transcriptional activity is highest and occurs mainly in the nonreproductive tissues surrounding the microspores, especially the tapetum. On the other hand, in microspores, a constant low level of *AtGA3ox3* and *AtGA3ox4* transcripts is observed, which remains through stages 11–12. At this time, a dramatic decrease in the mRNA content of both genes in anther walls is correlated with the subsequent degradation of the tapetum. Finally, *AtGA3ox3* and *AtGA3ox4* are only expressed in pollen grains up to and during dehiscence [36]. This discovery is consistent with results obtained from rice demonstrating that pollen develops the capacity for GA biosynthesis relatively late in the developmental process [49,51]. Based on the expression of *AtGA3ox1*, which is a paralogue of *AtGA3ox3* and *AtGA3ox4*, it was assumed that the filament is also a site of GA biosynthesis [36,50]. However, there is no expression of the single-copy early biosynthesis gene *AtCPS*, which is only expressed in anthers. This may suggest that GA precursors are transferred from anther to filament [30,52]. Further studies showed that mutations in different late GA biosynthesis genes affect the final phases of stamen development, even in stages after the degradation of the tapetum. In *A. thaliana*, the *ga20ox1 ga20ox2* and *ga3ox1 ga3ox3* double mutants exhibit disturbance of filament elongation as well as delayed or inhibited anther dehiscence [36,37]. Furthermore, the *ga20ox1 ga20ox2* mutant has fully viable pollen, whereas *ga3ox1 ga3ox3* exhibits defective pollen after its maturation in the floral stage 11/12 [36]. Thus, these defects are not related to pollen development but to stamen maturation and dehiscence [1].

All of the above results clearly demonstrate that GA biosynthesis and the presence of active GA molecules in the appropriate concentration at various stages are extremely important for the proper development and functioning of the male reproductive organs in several different plant species (Table 1).

### 3.2. Perception, Signal Transduction and Action of GAs during Stamen Development

#### 3.2.1. GA Signaling Pathway—General Information

GA signaling is received by the soluble receptor GA INSENSITIVE DWARF1 (GID1), which is located in both the cytoplasm and in the nucleus [54]. In the rice genome, a single *GID1* gene has been identified, while three of its orthologs, *GID1a*, *GID1b* and *GID1c*, are found in *A. thaliana* [35,54]. The binding of bioactive GAs to the receptor promotes its interaction with DELLA proteins (their name was created on the basis of a short stretch of amino acids D-E-L-L-A in their N-terminal region), which are the major repressors of the GA signaling pathway. The DELLA motif present in all DELLA proteins is important for this type of interaction, and its removal results in an inability to form the GID1-DELLA complex despite the presence of GAs. In rice and *Hordeum vulgare* (barley), one DELLA protein has been found [SLENDER RICE1 (SLR1) and SLENDER1 (SLN1), respectively], while in *A. thaliana*, five DELLAs have been identified [GA INSENSITIVE (GAI), REPRESSOR OF GA1-3 (RGA) and RGA LIKE1/2/3 (RGL1/2/3)], which belong to the GRAS family. The binding of DELLAs by the GA-GID1 complex increases their affinity for the SCF^SLY1^ E3 ubiquitin ligases in *A. thaliana* or SCF^GID2^ in rice, and polyubiquitin chain-labeled proteins are degraded in 26S proteasomes. These events lead to the activation/unblocking of specific transcription factors that interact with the promoters to regulate GA-target genes (Figure 4B) [45,46,47,48,49,50,51,52,53,54,55,56].

#### 3.2.2. Receptor—Dependent Signaling

GAs play a minor role in the very early stages (initiation) of stamen development. Phenotypic analyses of GA signaling mutants in rice and *A. thaliana* indicate that this phytohormone plays an important role in the development and functioning of the tapetum cell layer and pollen (microgametophyte), which are tightly intertwined [1]. Loss of the GID1 receptor in the *osgid1-4* rice mutant causes anther-wide developmental arrest to occur either just prior to or during meiosis. Then, pollen mother cells do not form tetrads. The exact cause of this phenomenon is unknown, but it unambiguously indicates that GID1-dependent signaling is required for the proper course of meiosis. It is assumed that the loss of GA signaling in tapetum cells may indirectly block the further development of the gametophyte, although GA signaling may act directly on pollen mother cells [48]. Expression analysis performed by Chhun et al. [49] showed that the transcriptional activity of *OsGID1* and other genes involved in GA signaling (*GID2*, *SLR1*, *GAMYB*) actively occurs at the premeiosis stage in the pollen development process. Consequently, the transmission of GA signaling genes occurs in a sporophytic manner [49]. Anther development in the *A. thaliana gid1a-1 gid1b-1 gid1c-1* triple receptor mutant has not been described, whereas the *gid1a-1 gid1b-1 gid1c-2* mutant is reported to be non-flowering [1]. The analysis of GA signaling mutants is additionally limited by functional redundancy. All three *AtGID1* genes act redundantly to promote stamen filament development. Loss of *AtGID1A* and *AtGID1B* specifically reduces filament elongation, but not to the extent seen in the triple loss-of-function mutant [35]. Moreover, the triple receptor mutant exhibits more pronounced disturbances in stamen development than *ga1-3*, and the phenotype of WT plants cannot be restored via GA application [1,46].

#### 3.2.3. The Role of DELLA Proteins

Extensive studies concerning the effect of GAs on microsporogenesis and pollen tube elongation in rice have shown that the semifertility of the *Slr1-d3* mutant (mutation located at the *SLR1* locus) is mainly caused by impaired pollen development rather than weakened pollen tube elongation, as demonstrated for the *rpe1* mutant [49]. As previously mentioned, the requirement for GA synthesis in rice pollen for pollen tube elongation directly indicates that the GA signaling pathway should be active in pollen. These differences in fertilization between the GA signaling and GA biosynthesis mutants are difficult to explain [49]. 

It has been suggested that DELLAs play a role in controlling stamen filament development by the observation that transgenic expression of WT or mutant forms of GAI can retard stamen elongation and induce male sterility in tobacco and *A. thaliana* [57,58]. Furthermore, mutants lacking the rice SLR1 or barley SLN1 protein also exhibit infertility due to impaired floral development [59,60]. In *A. thaliana*, mutants lacking GAI, RGA, and RGL2 alone or GAI and RGA together do not show suppression of the *ga1-3* floral phenotype, although *GAI*, *RGA*, *RGL1,* and *RGL2* are all expressed in developing inflorescences [61,62,63]. Additional genetic studies showed that RGA, RGL1, and RGL2 act synergistically in the repression of *A. thaliana* stamen and anther development in GA-deficient plants; nevertheless, the GA signal is mediated primarily by the RGA and RGL2 proteins with a small contribution from RGL1 [40]. The phenotype of the *ga1-3* mutant, in which GAI, RGA, RGL1, and RGL2 function is simultaneously lost (*ga1-3 gai-t6 rga-t2 rgl1-1 rgl2-1*), confirms that the mechanism of DELLA degradation is crucial for the proper development of both stamens and the entire flower [40,64]. Contrary to the single *ga1-3* mutant, the penta mutant can produce fertile flowers despite the lack of GAs due to the constant activation of the phytohormonal pathway related to a complete lack of DELLA repressors. Comparative analysis of the gene expression pattern among WT, *ga1-3* and *ga1-3 gai-t6 rga-t2 rgl1-1 rgl2-1* plants allowed the identification of 360 genes essential for flower development whose transcriptional activity is inhibited by DELLA (named for genes whose expression is downregulated in *ga1-3* but is restored to the WT level in the penta mutant) and 273 genes activated by these proteins (named for genes upregulated in *ga1-3* but restored to the WT level in the penta mutant) [64]. Interestingly, the results of transcriptome analyses involving DELLA-down and DELLA-up genes in the single and penta mutants during flower development and seed germination show significant differences, despite a similar impact of GAs on cells in both of the studied processes in *A. thaliana*. This observation shows that seed germination and floral development in this species are mediated by distinct DELLA-dependent GA-responsive transcriptomes [40,65]. Another study of RGA-induced global transcript changes in developing *A. thaliana* flowers showed that equal numbers of genes were up- and downregulated by RGA [66]. As might be anticipated, many of the downregulated genes are involved in metabolism, particularly that of cell walls. A very high proportion of these RGA-regulated genes are exclusively or predominantly expressed in stamens, indicative of the complexity of the processes regulated by GA signaling in anthers [30].

#### 3.2.4. Events Downstream of DELLAs during Filament Elongation and Anther Development

In *A. thaliana,* stamens have 34 genes whose transcriptional activity is inhibited by DELLAs been identified; among these genes, three are significant: *MYB21, MYB24,* and *MYB57* [67]. The MYB21 and MYB24 transcription factors belong to the 19^th^ subgroup of the R2R3-MYB family [68], and MYB57 exhibits high phylogenetic similarity to these transcription factors [69]. These three *MYB* genes are expressed at very low levels in the young flower buds of the *ga1-3* mutant, but their activity is restored to the level found in WT plants in the g*a1-3 gai-t6 rga-t1 rgl1-1 rgl2-1* penta mutant [67]. The detailed *MYB* expression studies in four quadruple mutants (*ga1-3 gai-t6 rgl1-1 rgl2-1*, *ga1-3 gai-t6 rgl1-1 rga-t2*, *ga1-3 rga-t2 rgl1-1 rgl2-1,* and *ga1-3 gai-t6 rga-t2 rgl2-1*) in which only one *DELLA* gene (*RGA* or *RGL2* or *GAI* or *RGL1*, respectively) was active indicated that only the RGA and RGL2 proteins, and not GAI or RGL1, are effective in inhibiting the expression of *MYB21*, *MYB24,* and *MYB57*. Those quadruple mutants with expression of only *RGA* or *RGL2* were completely sterile, unlike *GAI* or *RGL1*, which were fully fertile [46,67]. The question of whether *MYB* gene activity is essential and indispensable for proper flower development also seems important. To answer this question, Cheng et al. [67] performed a series of genetic tests mainly involving the generation of single, double, and triple *myb* mutants. The results of these studies revealed that *MYB21* plays a principal role in the control of filament elongation, while *MYB24* and *MYB57* function redundantly with *MYB21*. Mutations in the *MYB21* and *MYB24* genes influence the elongation of filamentous cells more than the proliferation of these cells (their number) [67]. The same situation applies to reduced filaments, the occurrence of which is observed in *ga1-3* mutants. Therefore, it can be concluded that MYB21 and MYB24 function downstream of DELLAs in the GA signaling pathway to control stamen filament growth [46].

As in the case of GA biosynthesis, in both *A. thaliana* and rice, the main site of GA action in the anther is the pollen and the surrounding tapetum cell layer. Pollen development is highly dependent on the tapetum, both for nutrition and the deposition of pollen wall components [18]. The results of microarray and mutant analyses indicate that the GA pathway may affect the function of the tapetum exclusively by regulating *OsGAMYB* in rice [48] or *AtMYB33* and *AtMYB65* in *A. thaliana* [70]. The phenotype of the *myb33 myb65* mutant is characterized by great hypertrophy of the tapetum and pollen abortion, and the similarity in phenotype to the corresponding rice mutant suggests a conserved function for GAMYB in tapetum development. Whereas no downstream targets of AtGAMYB proteins have been identified, OsGAMYB directly activates the expression of the lipid metabolism genes *CYTOCHROME P450 HYDROXYLASE* (*CYP703A3*), *β-KETOACYL REDUCTASE* (*KAR*), and *MALE STERILITY2* (*MS2*) involved in the synthesis of sporopollenin, which is an essential component of the Ubisch body in the tapetal cell and exine in the pollen coat [48]. This confirms the link between GA signaling and tapetum secretory functions. It should be noted that *A. thaliana* has a unique secretory tapetum that does not include Ubisch bodies, whereas most plants have a secretory tapetum that uses Ubisch bodies as a carrier material [48]. In rice, GA signaling is also necessary for the entry of the tapetum into the programmed cell death (PCD) pathway, although it is not clear whether this occurs directly or indirectly. A link with tapetum PCD has been established through the upregulation of *OsC6* by GAMYB binding [48]. *OsC6* also plays a role in rice tapetum secretory functions [71]. Both the *OsC6* gene encoding a lipid transfer protein and the *OsC1* gene encoding a cysteine protease are required for the entry of the tapetum into PCD and are regulated by *TAPETUM DEGENERATION RETARDATION* (*TDR*) [72], a putative rice homologue of *ABORTED MICROSPORES* (*AMS*) [3]. The bHLH transcription factor AMS [73] is a downstream target of another bHLH protein, DYSFUNCTIONAL TAPETUM1 (DYT1), which is itself a target of EXS/EMS [74]. Transcriptomic evidence suggests that OsGAMYB also regulates *TDR* expression [48]. It can be concluded that GAMYB regulates the stamen developmental program at multiple levels [1] (Table 2).

## 4. Jasmonate-Dependent Stamen Development and Functioning

Jasmonates are well-recognized stress hormones that regulate plant responses to biotic and abiotic stresses. An increasing number of studies have shown that JAs also function in a remarkable number of plant developmental events, including generative development [75]. The presence of jasmonic acid (JA) and its methyl derivative (MeJA) has been described in the anthers and pollen of three species of *Camellia*, while JA conjugates with isoleucine (JASMONOYL ISOLEUCINE, JA-Ile) in the pollen grains of *Pinus mugo* and *Petunia hybrida* [76]. JA involvement in the reproductive process became more evident when *A. thaliana* mutants incapable of responding to JA were shown to have a male-sterile phenotype. By analyzing these phenotypes, it was shown that JAs are important for proper pollen development and also exhibit interesting functions related to stamen elongation and the correct timing of pollen release [76,77]. Characteristic features related to stamen development of JA-deficient and JA-responsive mutants are presented in Table 3 and Table 4, respectively.

### 4.1. The Importance of JA Biosynthesis in Proper Stamen Development

#### 4.1.1. JA Metabolism—General Information

**Figure 5:**
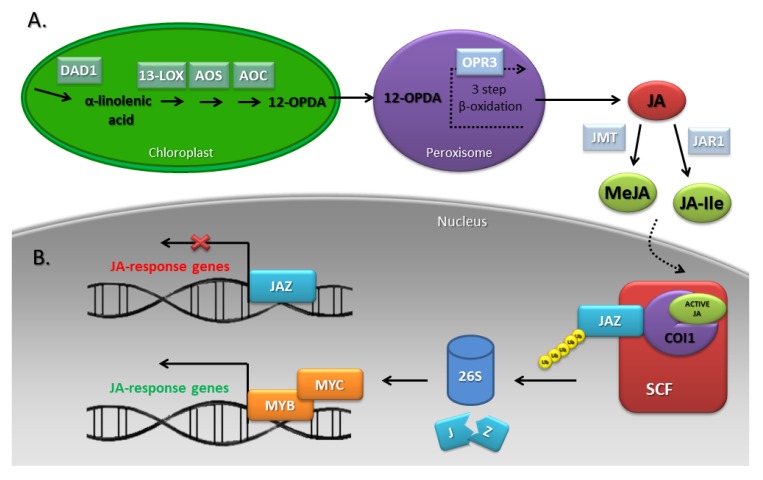
JA biosynthesis (**A**) and signaling (**B**) pathways. α-linolenic acid is released from membrane phospholipid by a lipolytic enzyme phospholipase A1 DEFECTIVE IN ANTHER DEHISCENCE1 (DAD1). Next, α-linolenic acid is converted to 12-oxophytodienoic acid (12-OPDA) by 13-lipoxygenase (13-LOX), allene oxide synthase (AOS) and allene oxide cyclase (AOC). Further conversions occur in the peroxisomes, where JA is formed in a reaction catalysed by oxophytodienoic acid reductase3 (OPR3) subjected to three-step β-oxidation. Finally, in the cytosol, JA is converted to MeJA by JA carboxy methyltransferase (JMT) or JA-Ile by jasmonate amino synthetase/jasmonate resistant1 (JAR1). Bioactive JAs interact in the nucleus with the CORONATINE-INSENSITIVE1 (COI1) receptor, which leads to the activation of the SCF ubiquitin ligase E3 and the degradation of JA ZIM-domain (JAZ) repressor in 26S proteasomes. This situation allows forming the MYC-MYB complex, which regulates JA-response genes [9,76].

The precursor of JAs is α-linolenic acid (α-LeA/18:3) released from chloroplast membranes with the participation of lipases encoded by genes such as the *DEFECTIVE IN ANTHER DEHISCENCE1* (*DAD1*) [78]. In chloroplasts, α-LeA is finally converted to 12-oxophytodienoic acid (12-OPDA) by 13-lipoxygenase (13-LOX), allene oxide synthase (AOS), and allene oxide cyclase (AOC) acting sequentially. Further transformations take place in the peroxisomes, where JA is formed in a reaction catalyzed by oxophytodienoic acid reductase3 (OPR3) subjected to three-step β-oxidation [76]. After transport to the cytoplasm, JA undergoes further metabolic changes to form MeJA or JA-Ile with the participation of JA carboxy methyltransferase (JMT) and jasmonate amino synthetase/jasmonate resistant1 (JAR1), respectively. JA-Ile can be inactivated by cytochrome P450 CYP94B3 [75,79] (Figure 5A). Genes encoding enzymes of the JA biosynthetic pathway are expressed specifically in such floral organs as the ovaries, petals and sepals. This floral organ-specific expression suggests that signals produced in these organs are transported, for example, to the stomium—the tissue responsible for the release of mature pollen grains [80]. Many studies conducted in *A. thaliana* for over 20 years have shown that JA-deficient mutants and lines overexpressing JA catabolism genes are male sterile due to the arrest of stamen development at anthesis [75]. 

#### 4.1.2. Studies on JA Biosynthesis Mutants

The *defective in anther dehiscence1* (*dad1*) mutant of *A. thaliana* shows defects in anther dehiscence, pollen maturation, and flower bud development. The *DAD1* gene encodes a particular chloroplast-specific phospholipase A1 (PLA1) lipolytic enzyme. Ishiguro et al. [78] demonstrated that before flower opening, all cell types (epidermis, endothecium, tapetum, immature pollen grains, connective) are normal in developing anthers of *dad1* mutant, similar to all structural features (disappearance of the tapetum, breakdown of the septum, differentiation of the stomium, development of fibrous bands in the endothecium and connective cells). Thus, there are no differences in structures between *dad1* and WT anthers before dehiscence, but they are interrupted immediately before stomium breakage. Additional investigation showed that the pollen grains of the *dad1* mutant develop normally up to the trinucleate stage; however, a defect occurs at the final stage of maturation contributing to inviability of the pollen grains. Another phenotype characteristic of the *dad1* mutant is the delay of flower bud opening, and importantly, the *dad1* mutant may be rescued by the application of JA as well as α-LeA. The expression profile of the *DAD1* gene is consistent with the defects associated with the *dad1* mutation [78]. In rice, the *P0491E01* gene, encoding a protein similar to DAD1 in *A. thaliana*, has been identified [81]. Cytological studies performed in male-sterile transgenic plants showed normal anther development at the initial stages, but microspore development into mature pollen grains was impaired. Additionally, the expression of the *P0491E01* gene was significantly reduced in transgenic plants, suggesting that JAs play an important role in later stages of anther development [76,81]. 

The *lox3 lox4* double mutant displays abnormal anther maturation and defective dehiscence. These plants are no longer self-fertile, and their pollen is not viable. Fertility in the double mutant can be restored genetically by complementation with either *LOX3* or *LOX4* cDNA or biochemically by the application of JA [82].

A screen for *A. thaliana* mutants with fertility difficulties in a transposon-tagged population allowed the identification of the *delayed dehiscence2-2* (*dde2-2*) mutant. This mutant exhibits problems related to the elongation of the filament and anther dehiscence. The WT phenotype was recovered by the application of MeJA, indicating that this mutation directly affects the JA biosynthesis pathway. Additional studies of gene expression and complementation analysis showed that *dde2-2* male sterility is caused by interruption of the gene sequence encoding ALLENE OXIDE SYNTHASE (AOS) [83].

A mutant for the *DELAYED DEHISCENCE1* (*DDE1*)/*OPR3* gene, which encodes a 12-oxophytodienoic acid reductase3 (OPR3), has also been recognized [19,84,85]. In this mutant, the cellular organization and differentiation of anther tissues appear normal, but dehiscence does not occur at flower opening, and pollen grains are inviable. In situ hybridization studies performed by Sanders et al. [84] showed that during the early stages of floral development, *DDE1* transcripts accumulate within all floral organs. Later, *DDE1* mRNA appears specifically within the pistil, petals, and stamen filaments. The *DDE1* transcript was not detected in the stomium and septum cells of the anther, which are involved in pollen release. Therefore, JAs play an important role in the timing of anther dehiscence [84]. *opr3* mutant plants are sterile, and they can be induced to become fertile by JA application but not by OPDA treatment. These results indicate that the signaling molecule that induces and coordinates anther filament elongation, the opening of the stomium, and the production of viable pollen is JA [85].

The results of the research presented above indicate that JAs mainly control late stages of male generative organ development in the 13th phase of flower development [85]. Obtaining new mutants for other important enzymes of the JA biosynthetic pathway will certainly increase our understanding of the complex mechanisms taking place during plant reproduction (Table 3).

### 4.2. Perception, Signal Transduction and Action of JAs during Stamen Development

#### 4.2.1. JA Signaling Pathway—General Information

Bioactive JAs interact in the nucleus with the CORONATINE-INSENSITIVE1 (COI1) receptor, an F-box protein (FBP), which leads to the activation of the SCF^COI1^ ubiquitin ligase E3 and, consequently, the proteolytic degradation of the JA pathway repressors—JA ZIM-domain (JAZ) proteins. Reduction of JAZ levels allows the release of the downstream IIIe bHLH MYELOCYTOMATOSIS ONCOGENES (MYC) and R2R3 MYB transcription factors to regulate JA-mediated stamen development (Figure 5B) [9,86]. 

#### 4.2.2. JA Signaling Dependent on the COI Receptor

In the *A. thaliana coi-1* mutant, abnormal elongation of the filaments, delayed anther dehiscence and reduced pollen viability in the 13th flower development phase are observed [46]. Restored *COI1* expression in the epidermis of the filaments and anthers can recover the development of these organs in the *coi1* background [87].

COI1 plays a similar role to an F-box protein in other plant species and might contribute to the regulation of stamen development. *A. thaliana* transgenic *coi1-1* plants expressing the soybean *GmCOI1* gene show a male fertile phenotype comparable to that of WT plants [88]. Identical results were obtained by overexpressing the *OsCOI1* gene from rice in *A. thaliana coi1-1* mutants [89]. Despite the similarities in the signal perception pathways of various plants, the JA response can be evolutionarily modified. The tomato *LeCOI1* gene is necessary to regulate responses to pathogens, while it is not responsible for male fertility, suggesting that in species evolutionarily distant from *A. thaliana* or rice, the development of the stamen may be controlled in a JA-independent manner [90].

#### 4.2.3. The Pathway Downstream of the JAZ Repressor

To gain better insight into the mechanism of stamen development controlled by JAs, Mandaokar et al. [91] analyzed the gene expression profile in whole stamens of the *opr3* mutant treated with or without JAs. On this basis, 821 genes specifically induced by these phytohormones and 480 genes whose expression was inhibited were identified. Furthermore, 13 of these genes whose transcriptional activity was closely related to stamen maturation were recognized, including *MYB21*, *MYB24,* and *MYB57* [91]. These three genes have already been described in the section on GA signaling as genes that are inhibited by DELLA repressors. The transcriptional approach adopted by Mandaokar et al. proved that *MYB21*, *MYB24*, and *MYB57* are involved in the JA signaling pathway as well. The analysis of mutant phenotypes showed that both the single *myb21* and the double *myb21 myb 24* mutants are characterized by very short filaments, delayed anther dehiscence, and greatly reduced male fertility, and it is impossible to restore the WT plant phenotype via JA application [91]. These results indicate that *MYB21* and *MYB24* are induced by JAs and mediate important aspects of this phytohormone response during stamen development. Additionally, the *opr3* mutant in which the *MYB21* gene was simultaneously overexpressed regained fertility and filament elongation occurred as in WT plants, whereas *MYB21* overexpression can restore stamen development in *coi1-1* plants [67,92]. Further investigation allowed the determination of the existence of another R2R3 MYB factor, MYB108, which regulates anther development [93]. *MYB108* expression is largely restricted to sporophytic tissues of the stamen. *myb108* exhibits reduced male fertility associated with delayed anther dehiscence and reduced pollen viability. It was also established that *MYB108* and *MYB24* exhibit overlapping functions and act downstream of *MYB21* in a transcriptional cascade that mediates stamen and pollen maturation in response to JAs [93].

Recent studies carried out by Qi et al. [94] showed that one of the MYC transcription factors—MYC5—acts as a target of JAZ repressors to function redundantly with other MYC factors such as MYC2, MYC3, and MYC4 in the regulation of stamen development and seed production [94]. All of these MYC TFs belong to the IIIe bHLH TF family and exhibit a special bHLH_MYC_N domain in the N-terminal region, a bHLH region in the C-terminus [95], and non-bHLH amino acid motif [96]. Moreover, these MYC factors interact with the MYB transcription factors MYB21 and MYB24 to form the MYC-MYB transcription complex and cooperatively regulate stamen development [94]. Stamens from *myc2 myc3 myc4 myc5* quadruple mutant plants at anthesis exhibit the same defects as *coi1-1* mutants, but they ultimately become fertile, resulting in partial male sterility. The expression of *MYB21, MYB24, MYB57,* and *MYB108* is repressed in the flowers of the *myc2 myc3 myc4 myc5* quadruple mutant. Overexpression of *MYC5* and *MYC3* can partially restore the expression of *MYB21, MYB24,* and *MYB57* in *coi1-1*, which affects stamen development and fertility. Moreover, the expression of *MYC2* is obviously increased in the flowers of the *myb21 myb24* double mutants, while the expression of *MYC5* is not elevated [94]. A previous study showed that *myb21 myb24* exhibits elevated JA biosynthesis in flowers [97]. It is likely that the elevated JA biosynthesis in *myb21 myb24* may upregulate the expression of *MYC2* but not that of *MYC5*. Consistent with this idea, Qi et al. [94] found that JA treatment significantly induced the expression of *MYC2* but not that of *MYC5*. The feedback regulation between the MYCs and MYBs is complicated and deserves further investigation [94]. 

Another study conducted by Chen et al. [98] revealed differences among MYC and MYB factors. *MYC2* and *MYC3* exhibited high expression in all floral organs, while *MYC4* and *MYC5* were preferentially expressed in sepals or carpels, respectively. Additionally, overexpression of MYB factors inhibits stamen development, while overexpression of MYC factors cannot influence stamen development. Furthermore, the expression of *MYB21, MYB24, MYC2, MYC3,* and *MYC4* can be either significantly or mildly induced by JA, whereas that of *MYC5* cannot. As these MYB and MYC factors share common functions and exhibit differences at the same time, it will be interesting to investigate what plant responses the MYC-MYB complex regulates in addition to stamen development, as well as what other plant responses the MYC and MYB factors control separately and in what way [98]. To answer these and other questions, we recommend the most recent article by Chen et al. [95]. In rice, *OsMYC2* is expressed in all tissues and is highly expressed in the spikelets and floral organs [99]. It regulates spikelet development through the interaction with OsJAZ1 and the activation of the downstream gene *OsMADS1* [100] (Table 4).

## 5. Gibberellin-Jasmonate Interactions in the Regulation of Stamen Development

Independent discoveries regarding the regulation of the transcriptional activity of the *MYB21*, *MYB24* and *MYB57* genes by GAs and JAs (also discussed in separate sections) prompted Cheng et al. [67] to verify whether the signaling pathways of these phytohormones act in parallel independently in the male sexual organ development or whether there is a certain hierarchy between them [67]. For this purpose: (1) JAs were applied to the flower buds of the *ga1-3 gai-t6 rga-t2 rgl1-1* mutant, lacking biosynthetic capacity and a GA response; and (2) GAs were applied to the JA biosynthesis mutant *opr3*. In the first case, increased expression of *MYB21, MYB24,* and *MYB57* in flower buds was observed, while in the other case, no such effect was noted. Therefore, it was concluded that the JA pathway acts downstream of the GA pathway in modulating the expression of the three *MYB* genes in one of two ways: (1) JAs can alter the stability or activity of DELLA proteins to induce *MYB* expression or (2) GAs contribute to the degradation of DELLAs, thus stimulating the production of JAs or modulating the JA pathway for the induction of *MYB* genes. The results of molecular analyses led to rejection of the first possibility, because in the *ga1-3 gai-t6 rga-t2 rgl1-1* mutant after JA application, neither an increased level of DELLAs nor elevated expression of GA response genes was found, although the abundance of *MYB21, MYB24* and *MYB57* transcripts was significantly increased. On the other hand, the fact that the JA application causes an increase in *MYB* mRNA in a mutant that is unable to synthesize and transmit GA signals strongly suggests that JA biosynthesis is partially blocked in *ga1-3 gai-t6 rga-t2 rgl1-1*. Therefore, the levels of JAs in the tissues of young flower buds in a quadruple mutant were measured and were found to be significantly lower compared to those in WT plants [67], which was confirmed by studies on the expression of numerous genes associated with JA biosynthesis. By repressing DELLAs, GAs mainly increase the levels of *DAD1* and *LOX1* transcripts, leading to an increase in JA production [67]. However, the question remains of whether the induction of JA biosynthesis by GAs is important and crucial for the stimulation of *MYB* transcriptional activity. Exogenous GAs applied to the GA biosynthesis and signaling mutant (*ga1-3 gai-t6 rga-t2 rgl1-1*) first enhances the expression of *DAD1*, followed by the activation of *MYB* genes [67]. Nevertheless, the way in which the GA-DELLA-dependent pathway controls the level of *DAD1* transcripts is still unclear. Research results from Yu et al. [103] and Ito et al. [104] indicate mediation by AGAMOUS (AG) protein, which can directly regulate *DAD1* activity [103,104]. Therefore, it will be of interest to study the relationship between the DELLA, AG and DAD1 proteins in the future. In addition, the data obtained by Cheng et al. [67] indicate reduced expression of *LOX1* in the *ga1-3* mutant and its restoration to the level observed in WT plants in the penta mutant [67]. In summary, it can be concluded that GAs are a key endogenous signal involved in the regulation of JA biosynthetic gene expression [105]. It should be noted, however, that the expression of *MYB21*, *MYB24,* and *MYB57* in the *ga1-3 gai-t6 rga-t2 rgl1-1* mutant after exogenous GA application is not sufficient to restore the phenotype of WT plants. Thus, the activity of three *MYB* genes is necessary but not sufficient to ensure correct flower development mediated by GAs. It has therefore been suggested that modulation of the JA pathway may be just one of many ways in which GAs control correct stamen development [46].

**Figure 6:**
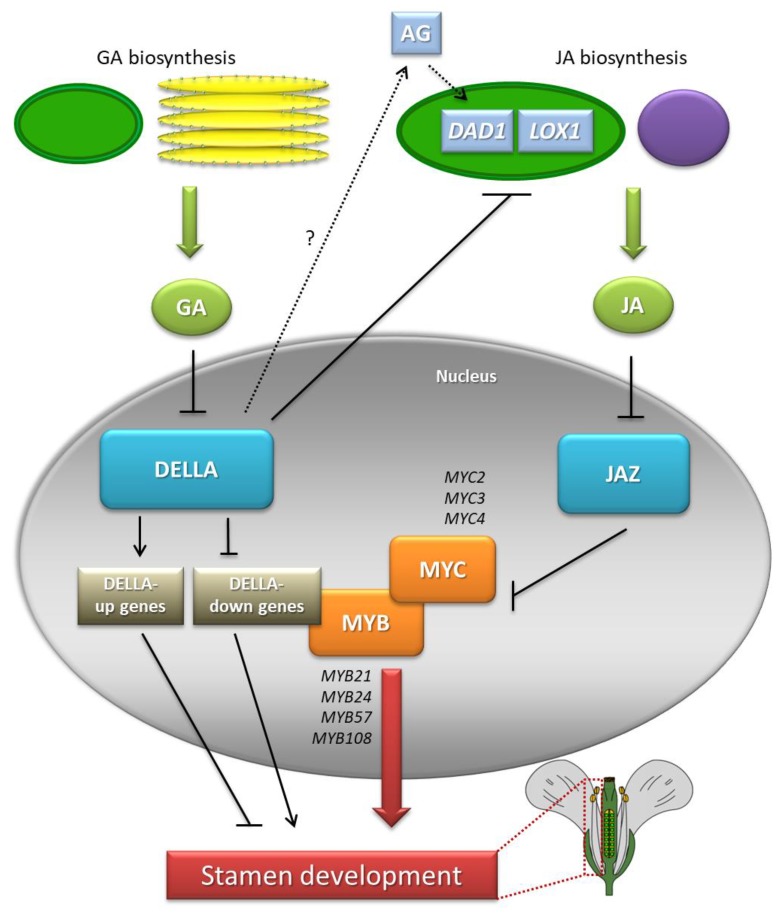
Interactions between GAs and JAs during stamen development in *A. thaliana*. GAs trigger the degradation of DELLAs to increase the expression of JA biosynthesis genes *DEFECTIVE IN ANTHER DEHISCENCE1* (*DAD1*) and *LIPOXYGENASE1* (*LOX1*), which promotes JA production. Degradation of JA ZIM-domain (JAZ) repressor induces the transcriptional activity of *MYC* and *MYB* genes (DELLA-down genes) for proper stamen development. GAs also act via a JA-independent pathway mediated by unknown GA-response factors. According to [46,75,94].

In connection with the discovery by Qi et al. [94] of a new mode of JA-regulated stamen development involving the *MYC-MYB* transcription factor complex, the relationship between JAs and GAs in this process can be expanded upon. The authors speculate that GAs also act through the MYC-MYB complex to regulate stamen development. DELLA proteins inhibit the expression of JA biosynthesis genes, thereby leading to the suppression of stamen development [94]. DELLAs directly interact with and repress MYC2 [106]. It will be interesting to investigate whether the DELLA proteins directly target all MYC and MYB members to inhibit the transcriptional function of this complex for the regulation of GA-mediated stamen development [94] (Figure 6).

## 6. Hormonal Transport in Stamen Development

Phytohormone transport plays a key role in the control of many physiological processes. The discovery of phytohormone transporters suggests that the movement of their molecules in the plant requires specialized proteins [107]. Recent studies have shown that NPF family proteins can act as phytohormone transporters: AIT3/NPF4.1 has the ability to transport ABA and GAs [108], while GTR1/NPF2.10, which preferentially transports glucosinolates [109], is able to transport GAs and JAs in the stamen. In vitro studies confirmed the ability to transport only active forms of GA_3_ and JA-Ile. However, the results of research on other transporters of this group indicate the possibility of post-translational modifications that may result in the ability to transport other forms. Interestingly, the *gtr1* mutant exhibits disturbances in anther dehiscence and filament elongation, resulting in male sterility [110]. The application of GA on the mutant resulted in the restoration of the correct phenotype. *GTR1* expression was mainly observed in the vascular bundle of the stamen filament in phases 13 and 14, which may suggest that GTR1 exports GAs from the vascular bundle to other cells to promote filament elongation [110]. In rice anthers, a very high concentration of GAs is observed, mainly in tapetum tissue, which is not observed in other parts of the flower [111]. This indicates that anthers may be the main site of GA biosynthesis in various parts of the flower; thus, there is a high demand for transport, which confirms the potential role of the GTR1/NPF2.10 transporter [110].

## 7. Summary

Proper stamen development in plants is fundamental to maintain the continuity of a species, and abnormalities in this process often lead to male sterility. This complicated process is subject to strict, precise genetic control, which determines the correct anatomy and morphology of the stamens, the production of viable pollen grains, and, as a result, the proper course of pollination and fertilization. In turn, plant hormones are the principal endogenous transducers of genetic information. Deficiencies in the biosynthesis and/or signal transduction pathways of GAs and JAs attenuate stamen development, pollen maturation or anther dehiscence. The results of numerous studies also indicate the involvement of other phytohormones in stamen development, e.g., auxins, BRs or CKs. Auxins mainly regulate the early phases of stamen initiation as well as the elongation of filaments. Additionally, the auxin response is intertwined with the JA regulation of late stamen development (pollen maturation and anther dehiscence). In turn, BRs control pollen grain production and filament elongation, and CKs are essential for anther dehiscence and pollen viability [9]. Thus, further studies on complicated networks of phytohormonal connections regulating the development and functioning of stamens are extremely important.

## Figures and Tables

**Table 1:** Stamen-related features of GA deficient mutants. *ent*-copalyl diphosphate synthase (CPS); *ent*-kaurene acid oxidase (KAO); GA 20-oxidase (GA20ox); GA 3-oxidase (GA3ox); *reduced pollen elongation1* (*rpe1*); pollen mother cells (PMCs).

Gene	Mutant	Phenotype	Species	Refs
*AtCPS*	*ga1-3*	Male-sterile phenotype, which can be reversed by GA application	*Arabidopsis*	[40]
		Anther and pollen development is blocked after meiosis but prior to mitosis		
		Pollen sacs expansion arrest		
		Inability to release microspores		
		Tapetum remains at the vacuolated stage and degenerates together with the microspores		
		Inhibition of filament elongation by a reduction of the length, not the number of cells		
		Altered ratio of stamen-pistil length in the flowers of mature mutant		
*LeCPS*	*gib-1*	Initiation of floral meristems and development of all floral organs proceeds normally up to a certain point, but then normal development ceases and flower buds eventually abort	tomato	[47]
		Microsporogenesis is blocked before meiosis		
		Anthers of developmentally arrested buds contain PMCs that are in the G1 phase of premeiotic interphase. Following treatment of mutant buds with GA_3_, premeiotic DNA synthesis and callose accumulation in PMCs are evident by 48 h posttreatment, and within 66 h, prophase I of meiosis and meiosis-related changes in tapetum development are observable		
*OsCPS1*	*oscps1-1*	One of the most severe GA-deficient mutant	rice	[48]
		GA treatment rescues the defect in stamen development		
		Abnormal enlargement of tapetal cells, to the point of almost filling the locule space		
		Collapse of microspores		
*LeKAO*	*ga-2*	Flower buds are initiated, but do not develop to maturity and eventually abort	tomato	[39]
		Cells of the sporogenous layer are initiated, but growth is arrested and cells eventually degenerate		
		Inhibition of microsporogenesis occurs before meiosis		
		Stamen do not elongate		
*OsKAO*	*rpe1*	Intermediate severity GA-deficient mutant	rice	[49]
		The mutant develops typical flowers with normal pistils and stamens		
		Pollen viability and the number of mature pollen grains in mutant are similar to those of the WT plant		
		Impaired pollen germination and elongation		
*GA20ox*	*ga20ox1* *ga20ox2*	Semidwarf, semifertile phenotype, with early flowers failing to set seed	*Arabidopsis*	[37]
		Self-rescue of seed set occurs in later flowers, although the mechanism remains undetermined		
		Normal tapetum degradation		
		Fully viable pollen		
		Delayed or inhibited anther dehiscence		
		Disturbance of filament elongation		
	*ga20ox1* *ga20ox2* *ga20ox3-1*	For many phenotypic characters, the triple mutant is not significantly different from the *ga1-3*		[53]
		Postmeiotic arrest in stamen development		
		Defect in tapetum degeneration. Tapetum layer fails to degenerate completely and remains in anther locules		
		Inhibited anther dehiscence		
		Do not undergo late-stage stamen acceleration, with growth and development instead halting		
		Shorter stamens at flower opening		
*GA3ox*	*ga3ox1 ga3ox3 *	The epidermal layer of the anther remains intact, although the tapetum layer disappears, suggesting that anther development is arrested around stages 11 and 12	*Arabidopsis*	[36]
		Defective pollen after its maturation		
		Delayed or inhibited anther dehiscence		
		Disturbances in filament elongation		
		All defects gradually decrease in the later flowers		

**Table 2:** Stamen-related features of GA responsive mutants. GA INSENSITIVE DWARF1 (GID1); *GA INSENSITIVE* (*GAI*); *REPRESSOR OF GA1-3* (*RGA*); *RGA LIKE1*/*2* (*RGL1*/*2*); *SLENDER RICE1* (*SLR1*); *SLENDER1* (*SLN1*).

Gene	Mutant	Phenotype	Species	Refs
*AtGID1*	*gid1a-1 gid1b-1 gid1c-1 *	Complete infertility and unresponsiveness to GA treatment	*Arabidopsis*	[35]
		The triple mutant exhibits more pronounced disturbances in stamen development than *ga1-3*		
		Anther development in this mutant has not been described		
		Dramatic reduction in length of filaments		
*OsGID1*	*gid1-4*	Anther-wide developmental arrest to occur either just prior to or during meiosis	rice	[48]
		PMCs are condensed and do not form tetrads		
		Abnormal stamens with shrunken and whitened anthers		
		Slightly enlarged tapetal cells that nearly fill the locule and contain the degraded meiocyte		
		Middle layer of cells does not degrade		
		Failure in epidermal cell expansion		
*GAI* *RGA* *RGL1* *RGL2*	*ga1-3 gai-t6 rga-t2 rgl1-1 rgl2-1*	Penta mutant can produce fully developed fertile flowers as the WT control	*Arabidopsis*	[40]
	*ga1-3 gai-t6 rgl1-1 rgl2-1* *ga1-3 gai-t6 rgl1-1 rga-t2*	Those quadruple mutants with expression of only *RGA* or *RGL2* are completely sterile		
		Mutants are effective in inhibiting the expression of *MYB21*, *MYB24* and *MYB57*		
	*ga1-3 rga-t2 rgl1-1 rgl2-1* *ga1-3 gai-t6 rga-t2 rgl2-1*	Those quadruple mutants with expression of only *GAI* or *RGL1* are fully fertile		
		Mutants are ineffective in inhibiting the expression of *MYB21*, *MYB24* and *MYB57*		
*SLR1*	*Slr1-d3*	Constitutive GA responce mutant is semifertile, even though it develops normal flowers with morphologically normal stamens and pistils	rice	[49]
		The anthers appear normal and produce a similar number of pollen grains as WT plants		
		High frequency of nonviable pollen		
	*slr1-1*	Sterile phenotype		[59]
		Impaired floral development		
*SLN1*	*sln*	Infertility due to impaired floral development	barley	[60]
*MYB21* *MYB24* *MYB57*	*myb21-t1 myb24-t1 myb57-t1*	Pollen is partial viable	*Arabidopsis*	[67]
		Short stamens are the main cause of the mutant sterility		
*MYB33* *MYB65*	*myb33 myb65*	Anthers are smaller than those in the WT plants and fail to produce pollen. The block in pollen development appears to be premeiotic occurring between anther stages 5 and 6	*Arabidopsis*	[70]
		During sixth stage of anther development when the PMCs begin to separate in a clearly defined locule and the tapetum begins to vacuolate, the mutant is similar, except that the tapetum begins to enlarge. Next, the tapetum expand to such an extent that there is no locule, and the PMCs have an irregular shape. Whereas microspores form in the locule of WT anthers and eventually form mature pollen, the tapetum of the mutant continues to expand until the contents collapse and degenerate. The expansion of the tapetum appears to be due to an increase in cell size, not in cell number		
		Stamens shorter than their WT counterparts and fail to fully extend to the pistil		
		Other than sterility and the associated characteristics of sterile plants, mutant shows no obvious morphological differences from WT plants		

**Table 3:** Stamen-related features of JA-deficient mutants. Phospholipase A1 (PLA1); DEFECTIVE IN ANTHER DEHISCENCE1 (DAD1); 13-lipoxygenase (13-LOX); ALLENE OXIDE SYNTHASE (AOS); *delayed dehiscence2-2* (*dde2-2*); 12-OXOPHYTODIENOIC ACID REDUCTASE3 (OPR3); *delayed dehiscence1* (*dde1*).

Gene	Mutant	Phenotype	Species	Refs
*PLA1/* *DAD1*	*dad1*	WT phenotype can be rescued by the JA application	*Arabidopsis*	[76]
		Developmental delay of flower bud opening		
		Before flower opening, all cell types are normally developed in mutant anthers, similar to all structural features		
		Pollen grains develop normally up to the trinucleate stage		
		A defect in pollen grains occurs at the final stage of their maturation		
		Defective in anther dehiscence		
*P0491E01*		Normal anther development at the initial stages	rice	[81]
		Microspores development into mature pollen grains is impaired		
*LOX3* *LOX4*	*lox3 lox4*	Male sterile. JA application restored fertility	*Arabidopsis*	[82]
		Abnormal anther maturation		
		Pollen is not viable		
		Defective dehiscence		
		Shorter filaments		
*AOS*	*dde2-2 *	Male-sterile phenotype which can be rescued by Me-JA application	*Arabidopsis*	[83]
		Impaired anther dehiscence and filament elongation		
*DDE1/* *OPR3*	* dde1/opr3*	WT phenotype can be rescued by the MeJA application	*Arabidopsis*	[84,85]
		Floral organs develop normally within the closed bud		
		The anther locules do not dehisce at the time of flower opening		
		Pollen develops to the trinucleate stage		
		Pollen grains are predominantly inviable		
		The filaments do not elongate sufficiently to position the locules above the stigma at anthesis		

**Table 4:** Stamen-related features of JA responsive mutants. CORONATINE-INSENSITIVE1 (COI1); MYELOCYTOMATOSIS ONCOGENES (MYC).

Gene	Mutant	Phenotype	Species	Refs
*COI1*	*coi-1 *	Delayed anther dehiscence	*Arabidopsis*	[101,102]
		Reduced pollen viability in the 13th phase of flower development		
		Abnormal filament elongation		
*MYC2* *MYC3* *MYC4* *MYC5*	*myc2* *myc3* *myc4* *myc5*	Pollen grains do not germinate in vitro		[94]
		The anthers dehisce and release viable pollen at floral stage 15		
		The filament does not elongate normally at floral stage 13		
*MYB21* *MYB24*	*myb21* *myb24*	Greatly reduced male fertility. Restore the WT phenotype via JA application		[91]
		Delayed anther dehiscence		
		Very short filaments		
*MYB108*	*myb108*	Reduced male fertility		[93]
		Delayed anther dehiscence		
		Reduced pollen viability		
